# Improving respiratory disease detection through SSL-enhanced acoustic analysis and exercise-rest measurements

**DOI:** 10.3389/fmed.2026.1864436

**Published:** 2026-06-24

**Authors:** Álvaro Vera-López, Darío Tilves-Santiago, José Manuel Ramírez-Sánchez, Laura Docío-Fernández, Carmen García-Mateo, María Bustillo-Casado, Alejandro García-Caballero

**Affiliations:** 1 Multimedia Technologies Group (GTM), atlanTTic Research Center, Universidade de Vigo, Vigo, Spain; 2 Multimedia Technologies Group (GTM), Galicia Sur Health Research Institute (IIS Galicia Sur), Vigo, Spain; 3 Department of Internal Medicine, Complexo Hospitalario Universitario de Ourense, SERGAS, Ourense, Spain; 4 Department of Psychiatry, Complexo Hospitalario Universitario de Ourense, SERGAS, Ourense, Spain

**Keywords:** acoustic biomarker, cough and sustained vowel analysis, feature fusion, Long COVID, machine learning, physiological stress test, respiratory disease screening, Self-Supervised Learning (SSL)

## Abstract

**Background:**

Voice analysis has emerged as a promising non-invasive approach for monitoring respiratory and systemic health conditions. However, subtle physiological alterations are often difficult to capture using recordings collected at rest. In addition, combining traditional acoustic descriptors with modern self-supervised speech representations may provide complementary information for clinical voice analysis.

**Objectives:**

This study evaluates a generalized screening model integrating stress-induced acoustic analysis with machine learning. We investigate how physical exertion and the fusion of traditional acoustic features with self-supervised learning embeddings (such as wav2vec 2.0 and WavLM) enhance the diagnostic sensitivity of vocal and respiratory signals. Post-Acute Sequelae of SARS-CoV-2 (PASC) is used as a case study to evaluate the proposed framework.

**Methods:**

Utilizing the DICOPERIA-Voice dataset (*n* = 154), we collected recordings of sustained vowel phonation (/a/) and voluntary coughing at two clinical moments: resting state and following a physiological stress protocol (six-minute walk and one-minute sit-to-stand tests). We employed a dual-feature extraction strategy, combining traditional acoustic biomarkers with high-dimensional Self-Supervised Learning (SSL) embeddings from wav2vec 2.0, WavLM and HuBERT. Binary classification (PASC vs. Healthy) was performed using Logistic Regression, evaluated via stratified 5-fold cross-validation.

**Results:**

Physical exertion significantly improved classification performance and reduced model variability across all tasks. The fusion of acoustic features, WavLM and wav2vec 2.0 achieved peak F1-scores of 82.2% for vowel phonation and 80.8% for coughing both in post-exercise conditions. A cross-task late fusion model aggregation reached the highest overall performance, with an F1-score of 87.7%.

**Conclusion:**

Incorporating Self-Supervised Learning representations into acoustic analysis improves the sensitivity of voice-based screening, while post-exercise measurements further enhance the robustness and consistency of classification. Together, these strategies provide a scalable and objective framework for detecting respiratory and vocal sequelae in chronic or post-viral conditions. With further validation, this approach could be integrated into routine functional assessments, offering a rapid, non-invasive adjunct to clinical decision-making.

## 1 Introduction

Respiratory diseases represent a major global health challenge, often leading to persistent functional impairments that affect multiple physiological systems (1, 2). These conditions can disrupt normal respiratory mechanics, including airflow, lung capacity, and muscular coordination, which are essential not only for breathing but also for voice production (3). As a result, respiratory dysfunction can negatively impact the phonatory system, leading to voice disorders characterized by structural, neurological, or systematic impairments that disrupt normal phonation (4). Dysphonia, a frequent manifestation of voice disorders, may present as alterations in vocal quality, pitch and loudness, often leading to reduced intelligibility (5). The heterogeneous nature of dysphonia -arising from various conditions, ranging from minor physical lesions to severe nerve disorders- poses challenges for diagnosis, monitoring, and treatment (6, 7). This complexity highlights a critical need for robust, objective analytical approaches that can accurately characterize vocal alterations in diverse patient populations (8). In this context, recent studies suggest that voice signal analysis and Machine Learning (ML) are promising non-invasive tools for screening pathologies that impair the respiratory (9) and central nervous systems (10).

The global health impact of the COVID-19 pandemic has profoundly illustrated these challenges through Post-Acute Sequelae of SARS-CoV-2 (PASC), commonly known as Long COVID (11). PASC encompasses a heterogeneous set of symptoms that persist long after the acute phase of infection, including fatigue, dyspnea, chest pain, cognitive difficulties, and mood changes (12). These persistent manifestations have a considerable impact on the quality of life of affected individuals. Current clinical data indicate that approximately 25% of patients experience dysphonia during acute infection, and nearly 70% of those affected continue to suffer persistent voice sequelae after recovery (13). By focusing on PASC as our primary case study, our aim is to address a critical gap in long-term post-viral care: the lack of non-invasive, scalable tools to monitor respiratory recovery and vocal health.

Traditional clinical assessments rely on resting-state recordings, which may fail to reveal latent pathological markers (14). A core premise of this research is that the diagnostic sensitivity of voice signals is not static; it can be significantly amplified by subjecting the vocal and respiratory systems to a controlled stress test. Physical exertion serves as a clinical provocateur (15, 16) challenging the respiratory and phonatory subsystems to reach their full functional capacity. By utilizing recordings captured both before and after this physiological challenge, the present study evaluates whether post-exercise voice signals reveal more distinctive markers of impairment than baseline measurements.

The evolution of feature extraction is equally critical to advancing objective assessment. Traditionally, the field has relied on handcrafted acoustic features focusing on temporal, spectral, and energy domains (17). These features have long provided the foundation for objective assessment as they provide valuable physiological insights. However, their reliance on steady-state assumptions often limits their applicability. Modern Self-Supervised Learning (SSL) models –such as wav2vec 2.0 (18), WavLM (19), and HuBERT (20)- are pre-trained on massive unlabeled datasets to learn the underlying latent structure of speech signals and offer an alternative, high-dimensional representation of speech (21). By leveraging high-dimensional embeddings, these SSL representations complement traditional methods by providing data-driven representations of speech signals that may contain discriminative information beyond conventional manual feature engineering.

This work investigates the interplay between task conditions (resting state vs. post-exercise) and feature extraction methods (handcrafted acoustic features and SSL embeddings). Utilizing recordings of sustained phonation and coughing, we employ ML classifiers to determine how physical exertion and advanced SSL representations enhance the detection of respiratory and vocal impairments. While it is clinically plausible that physical exertion may exacerbate respiratory and vocal impairments associated with PASC, the role of post-exercise acoustic analysis in capturing these changes and supporting objective assessment remains an area requiring further study. In addition, the magnitude and consistency of the post-exercise effect across different vocal tasks, as well as its interaction with modern speech representations, remain incompletely characterized. Using PASC as a primary case study, our objective is to validate these methodological advances and to evaluate whether a controlled physiological stress protocol can enhance disease-related acoustic markers. This work also aims to support the development of objective and scalable screening strategies that complement self-reported symptoms and conventional resting-state examinations, with potential relevance for future clinical workflows.

## 2 Materials and methods

This study uses the DICOPERIA dataset, a voice dataset collected within the diagnostic component of the COPERIA project (22). This component aimed to improve the differentiation between patients with PASC and individuals who had fully recovered from COVID-19, and DICOPERIA was developed to support this objective. Building on this dataset, we investigate the diagnostic sensitivity of voice signals under different physiological conditions by comparing resting and post-exercise recordings using both traditional acoustic features and high-dimensional SSL embeddings.

### 2.1 Dataset

The study was registered in the U.S. Clinical Trials Registry under the code NCT05629793. The recruitment process was scheduled to begin on December 14, 2022, and concluded in November 2023.

#### 2.1.1 Study population

Participants were recruited within the COPERIA project in collaboration with the Persistent COVID Unit at Ourense Hospital and local primary care centers in Galicia. The cohort consists of 154 participants recruited between December 2022 and November 2023, categorized into two groups: a control group of 70 individuals who achieved full functional recovery from COVID-19 and a PASC group of 84 individuals experiencing persistent symptoms (e.g., fatigue, dyspnea) for at least three months post-infection.

The study population exhibits a deliberate gender imbalance, with 103 females and 51 males, reflecting the higher clinical incidence of PASC in women. Age distribution is primarily concentrated in the 40–60 range, accounting for approximately 70% of the participants.

#### 2.1.2 Data acquisition protocol

To evaluate the impact of physiological stress on vocal markers, voice signals were captured at two distinct clinical moments:

1. **Baseline** or **Resting**: Recordings were acquired with the participant in a seated position, refraining from any physical or medical exertion.
2. **H1** or **Fatigue**: This recording was obtained after the participant had completed a series of physical exercises designed to induce fatigue. The exercises included:
  - Six-minute walk test: Participants performed a straight walk for 6 minutes, following the protocol described in the reference study by Enright P.L. (23).
  - One-minute sit-to-stand: Participants sat in an armless chair and executed as many sit-to-stand actions as possible within a 1-minute duration, without employing their upper limbs. The procedure for this exercise was based on the study (24).

At each stage, participants performed three repetitions of two specific vocal tasks: the sustained phonation of the vowel /a/ and voluntary coughing. These tasks were selected to provide information on both airway dynamics and vocal fold stability. Recordings were acquired in a controlled clinical environment designed to minimize external acoustic interference, where only the participant's voice was recorded and healthcare personnel avoided speaking during acquisitions. All recordings were stored as mono-channel audio signals at a sampling rate of 48 kHz.

### 2.2 Feature extraction

To characterize respiratory and phonatory alterations associated with PASC, we adopted a dual representation strategy that integrates physiologically interpretable acoustic biomarkers and high-dimensional SSL embeddings. Our approach is based on the hypothesis that acoustic descriptors and SSL-derived representations capture complementary aspects of the voice signal. The two feature paradigms are described below:

1. **Acoustic features:** A comprehensive set of acoustic features was extracted to quantify the physical properties of the voice signals. Prior to feature extraction, recordings were resampled to 16 kHz. A voice activity detection procedure based on energy thresholding was applied to remove silent and non-vocal segments. The resulting signals were subsequently processed using pre-emphasis filtering (coefficient = 0.97) and amplitude normalization. The feature set included Mel Frequency Cepstral Coefficients (**MFCCs**), alongside subsets of Low-Level Descriptors based on *ComParE 2016* presented in (25). All features were extracted using a 25 ms window and 10 ms hop size at a 16 kHz sampling rate. For the MFCCs, 13 coefficients were computed using 40 Mel filters over the 0-8 kHz frequency range, with pre-emphasis (0.97), as well as delta and delta-delta coefficients, included to capture temporal dynamics. From the ComParE 2016 set, we selected **energy-based descriptors** to quantify signal intensity and noise levels; **voicing features** (including jitter, shimmer, fundamental frequency, and harmonics-to-noise ratio) to assess vocal fold stability and periodicity; **basic spectral descriptors** such as roll-off, centroid, and spectral entropy to analyze frequency distribution; and **RASTA-filtered spectral features** (Relative spectral transform - perceptual linear predictive) were extracted to represent smoothed auditory band energies. To adapt these variable-length frame sequences for classification, we implemented a statistical aggregation strategy. For each recording, the frame-level descriptors were condensed into a fixed-dimension vector by computing 11 statistical and distributional descriptors: mean, standard deviation, minimum, maximum, spectral entropy, skewness, kurtosis, and four quartiles (25th, 50th, 75th, and 90th percentiles). The resulting aggregated matrix was then flattened into a single 1D feature vector to ensure a uniform input for the classifiers.
2. **SSL embeddings:** In addition to traditional metrics, the study leverages high-dimensional embeddings from the following pre-trained SSL models: wav2vec 2.0, WavLM, and HuBERT. WavLM and HuBERT embeddings were obtained from models fine-tuned on the PC-GITA pathological speech corpus (26), while wav2vec 2.0 was used in its general pre-trained form (27). Although these models utilize different pre-training objectives, they share a common Transformer-based architecture capable of mapping raw waveforms into context-aware latent spaces. By learning from massive speech corpora, these models act as powerful feature extractors that provide high-dimensional representations of speech signals and have demonstrated strong performance in pathological speech classification tasks (28). All SSL models operated on audio resampled to 16 kHz. Wav2vec 2.0 embeddings were extracted using the general pre-trained XLS-R 300M model, with representations from the final layer globally averaged across time to yield a fixed 1024-dimensional embedding. For WavLM and HuBERT, embeddings were obtained from a learnable weighted combination of all transformer layers, with the resulting frame-level representations aggregated over time using an attention-pooling layer to produce a fixed 768-dimensional pre-classifier embedding for each recording.

Prior to model input, feature normalization is applied to ensure that all variables are on comparable scales, thereby improving training stability and mitigating the risk of any single feature disproportionately influencing the model. For each cross-validation fold, this standardization is performed by extracting the feature-wise mean and standard deviation strictly from the training dataset.

To assess the complementary diagnostic value of both paradigms, we implemented a feature fusion strategy in which acoustic features were concatenated with different combinations of SSL embeddings (e.g., Acoustic + WavLM + wav2vec 2.0). This integration constitutes a central contribution of the present study. While acoustic descriptors provide well-established physiological markers (e.g., jitter, shimmer, spectral energy distribution), SSL embeddings provide high-level context-aware representations that complement acoustic features through data-driven characterization of the speech signal. By unifying these representations into a single feature space, our aim is to construct a more complete respiratory and vocal profile of each participant.

A detailed summary of the feature dimensionality and statistical aggregation procedure is provided in the Supplementary Tables 1–3.

### 2.3 Classification and evaluation

**Figure 1:**
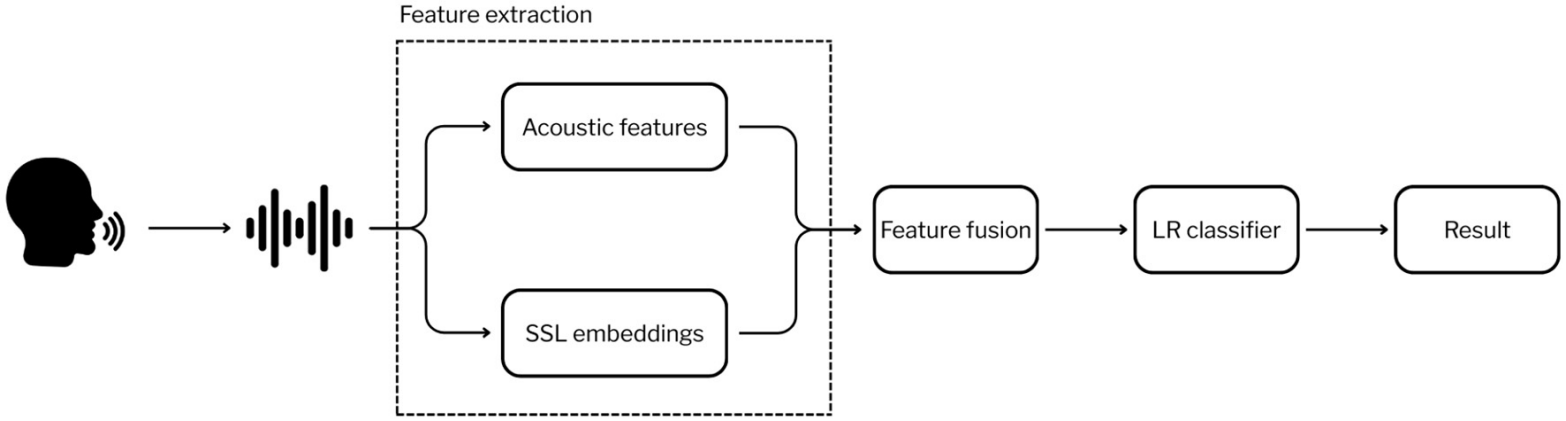
Overview of the proposed methodology.

Following extraction of acoustic features and SSL embeddings from cough and sustained phonation recordings, the resulting feature vectors were utilized as input for binary classifiers. The overall experimental workflow, encompassing feature extraction, and classification, is illustrated in Figure 1. As shown in the figure, each raw voice recording is first processed through two complementary feature extraction branches: one dedicated to acoustic descriptors and the other to SSL-based embedding extraction. These representations are generated independently from the same waveform and, depending on the experimental configuration, are evaluated separately or combined in a feature fusion stage through vector concatenation. The resulting feature set is subsequently provided to the classifier to discriminate between PASC and Healthy participants.

The classification task was framed as a binary problem (PASC vs. Healthy). To evaluate the interplay between physiological stress and feature representation, the dataset was analyzed across **four experimental subsets: Pre-Exercise and Post-Exercise recordings for both cough and sustained phonation tasks**.

For all experiments in this study, we employed a Logistic Regression (LR) classifier. The choice of a linear model was deliberate, as its high interpretability allows a direct comparison of the discriminative power inherent in the different feature sets and clinical conditions. LR was configured with L2 regularization and a fixed inverse regularization strength of *C* = 0.1, deliberately set below the default value of 1.0 to impose stronger penalization and mitigate overfitting in the high-dimensional, low-sample-size regime of this study. The classification pipeline was developed in *Python* 3.11, primarily using the *scikit-learn* library for model training and evaluation. The implementation used in this study is publicly available at https://github.com/GTMVigo/GTM-ssl-enhanced-respiratory-disease-detection.

Model performance was evaluated using subject-independent stratified 5-fold cross-validation, ensuring that all recordings belonging to a given participant were assigned exclusively to either the training or the validation set within any given fold, while preserving the original class distribution across folds. In each iteration, four folds were used for training and one fold was held out for validation, with the procedure repeated until each fold served as the validation set once. As each participant performed several repetitions of each task across clinical moments, the features extracted from all recordings belonging to the same participant were aligned chronologically and concatenated into a single participant-level representation before classification, with missing repetitions padded with zeros. Consequently, model training, inference, and the final performance metrics were executed and evaluated directly at the participant level.

Classification performance was quantified using a comprehensive set of evaluation metrics, including accuracy, precision, recall and F1-score. These metrics were computed from the number of true positives (TP), true negatives (TN), false positives (FP), and false negatives (FN), according to the definitions Equations 1–4:

$$\begin{array}{l} Accuracy=\frac{TP+TN}{TP+TN+FP+FN} \end{array}$$ (1)

$$\begin{array}{l} Precision=\frac{TP}{TP+FP} \end{array}$$ (2)

$$\begin{array}{l} Recall=\frac{TP}{TP+FN} \end{array}$$ (3)

$$\begin{array}{l} F1\text{-}score=2\cdot \frac{\text{Precision}\cdot \text{Recall}}{\text{Precision}+\text{Recall}} \end{array}$$ (4)

## 3 Results

This section presents a systematic analysis of the experimental findings, evaluating the classification performance across the four defined subsets of the DICOPERIA-Voice dataset. Although multiple architectures and feature configurations were evaluated during the experimental phase, we report here the comparison between the traditional acoustic baseline and the best-performing fusion model. Results are analyzed according to the two primary variables of study: the physiological recording condition (resting vs. post-exercise) and the nature of the feature representation (acoustic features and SSL embeddings).

Performance is evaluated using a comprehensive set of metrics, including accuracy, recall, and precision. However, results are reported and compared primarily using the F1-score, as it provides a balanced harmonic mean of precision and recall, ensuring a robust assessment in the presence of any class imbalance. Additionally, the Standard Deviation (STD) across the 5-fold cross-validation is reported to assess model stability and the consistency of the biomarkers across different data partitions.

### 3.1 Sustained vowel /a/ phonation results

The performance for the sustained phonation of the vowel /a/ is summarized in Table 1. Traditional acoustic features established a baseline with an F1-score of 66.7 ± 4.6% at rest, accompanied by an accuracy of 66.2 ± 4.8%, a recall of 66.4 ± 4.3%, and a precision of 66.9 ± 5.0%. Following physical exertion, the discriminative power of these acoustic biomarkers increased to an F1-score of 70.7 ± 5.6%. This improvement was consistent across the remaining metrics, with accuracy rising to 70.8 ± 5.8%, recall to 70.6 ± 5.9%, and precision to 70.8 ± 5.3%. These results suggest that the diagnostic threshold of traditional acoustic biomarkers is significantly improved when the respiratory system is pushed toward its functional limit.

**Table 1:** Classification performance for the sustained vowel /a/ phonation task.

Feature set	Audio moment	F1-score (%)	Accuracy (%)	Recall (%)	Precision (%)
Acoustic	Before	66.7 ± 4.6	66.2 ± 4.8	66.4 ± 4.3	66.9 ± 5.0
	After	70.7 ± 5.6	70.8 ± 5.8	70.6 ± 5.9	70.8 ± 5.3
Acoustic + wav2vec + wavlm	Before	78.3 ± 12.7	78.5 ± 12.5	78.2 ± 12.8	78.4 ± 12.6
	After	82.2 ± 4.7	81.9 ± 5.3	82.1 ± 5.1	82.2 ± 4.4

Results compare the acoustic baseline against the best-performing fusion model (Acoustic + wav2vec 2.0 + WavLM) across two physiological conditions: resting state (before) and post-exercise (after). Metrics reported as mean ± STD across 5-fold cross-validation.

**Figure 2:**
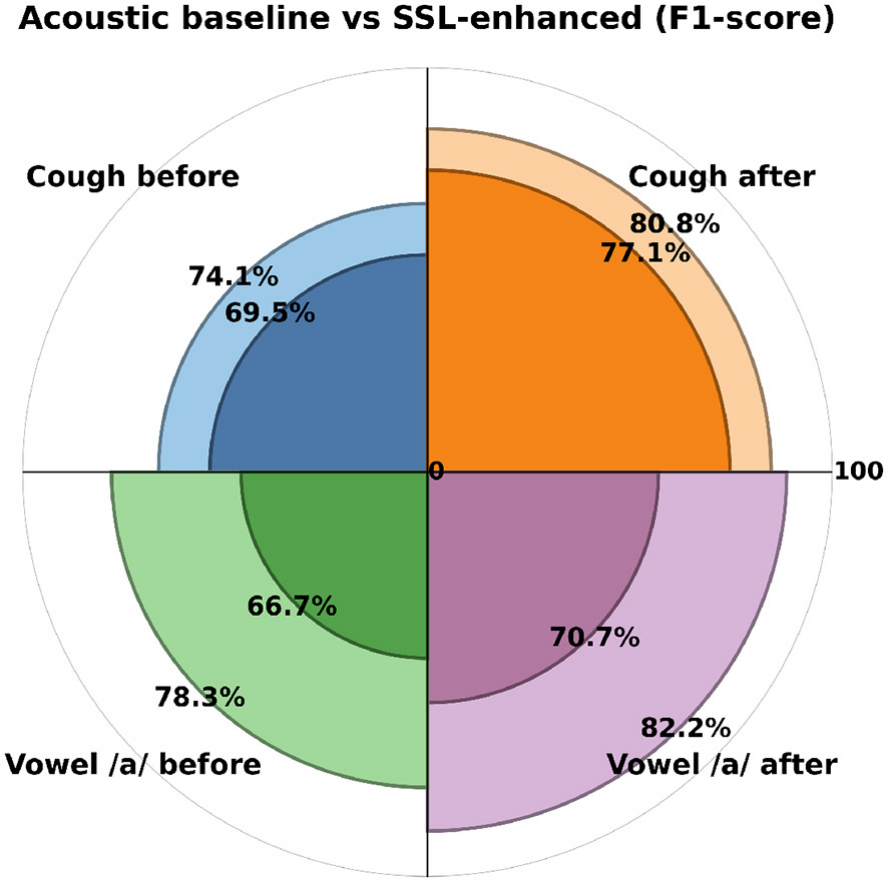
Comparative analysis of F1-scores between the acoustic baseline (inner lobes) and the SSL-enhanced fusion model (outer lobes). The radial bars represent the classification performance across tasks (cough and vowel /a/) and clinical moments (resting vs. post-exercise).

The integration of SSL-based high-dimensional embeddings significantly enhanced the model's discriminative capacity. The combination of acoustic features with wav2vec 2.0 and WavLM yielded the highest performance under post-exercise condition. At rest, this fusion achieved an F1-score of 78.3 ± 12.7%, together with an accuracy of 78.5 ± 12.4%, a recall of 78.2 ± 12.8%, and a precision of 78.4 ± 12.6%. After exercise, performance improved further to an F1-score of 82.2 ± 4.7%, with an accuracy of 81.9 ± 5.3%, a recall of 82.1 ± 5.1%, and a precision of 82.2 ± 4.4%. This represents an absolute increase of 3.9% in the F1-score due to physiological stress, paralleled by comparable gains in accuracy (3.4%), recall (3.9%) and precision (3.8%). This trend is illustrated in Figure 2, which compares the acoustic baseline and SSL-enhanced models across both tasks and recording conditions. Furthermore, the STD for this feature set decreased markedly after exertion. For the F1-score, the STD was reduced from 12.7% (before) to 4.7% (after). A similar stabilization effect was observed for accuracy (12.4% to 5.3%), recall (12.8% to 5.1%), and precision (12.6% to 4.4%). This reduction in STD indicates that physical exertion helps to homogenize the pathological markers of PASC, leading to more stable classification performance across different folds.

To further contextualize the benefits of this feature integration, Table 2 reports the classification performance of individual SSL models used in isolation without acoustic feature fusion. For this sustained vowel task, wav2vec 2.0 alone achieved an F1-score of 80.4 ± 6.6% at rest and 74.2 ± 2.5% after exercise, while WavLM alone reached 78.1 ± 2.0% at rest and 74.4 ± 15.0% post-exercise. Notably, while the standalone mean performance of these models appears highly competitive in the resting state, they exhibit high STD across cross-validation folds–particularly WavLM after exercise. In contrast, the fusion model consistently surpasses individual representations under post-exercise conditions while providing a markedly lower STD, confirming that feature concatenation delivers a more stable and reliable classification framework.

**Table 2:** Classification performance for the sustained vowel /a/ phonation task using individual SSL feature sets (WavLM and wav2vec 2.0) across resting state (before) and post-exercise (after) conditions.

Feature set	Audio moment	F1-score (%)	Accuracy (%)	Recall (%)	Precision (%)
wavlm	Before	78.1 ± 2.0	77.3 ± 2.3	77.5 ± 1.8	78.8 ± 2.8
	After	74.4 ± 15.0	74.1 ± 15.5	74.5 ± 15.1	74.3 ± 14.8
wav2vec	Before	80.4 ± 6.6	79.8 ± 7.1	80.3 ± 6.6	80.6 ± 6.7
	After	74.2 ± 2.5	74.0 ± 2.3	74.1 ± 2.5	74.4 ± 2.7

Metrics are reported as mean ± STD across 5-fold cross-validation and expressed as percentages.

### 3.2 Cough results

The classification performance for the coughing task followed a similar trend, as shown in Table 3. The baseline acoustic representation achieved an F1-score of 69.5 ± 10.8% before exercise, accompanied by an accuracy of 68.9 ± 10.2%, a recall of 69.4 ± 10.9%, and a precision of 69.5 ± 10.7%. After physical exertion, the performance improved to an F1-score of 77.1 ± 6.1%. This improvement was consistent across the remaining metrics, with accuracy increasing to 76.7 ± 6.1%, recall to 76.7 ± 6.1%, and precision to 77.4 ± 6.2%, representing absolute gains of 7.6% in F1-score, 7.8% in accuracy, 7.3% in recall, and 7.9% in precision.

**Table 3:** Classification performance for the voluntary cough task.

Feature set	Audio moment	F1-score (%)	Accuracy (%)	Recall (%)	Precision (%)
Acoustic	Before	69.5 ± 10.8	68.9 ± 10.2	69.4 ± 10.9	69.5 ± 10.7
	After	77.1 ± 6.1	76.7 ± 6.1	76.7 ± 6.1	77.4 ± 6.2
Acoustic + wav2vec + wavlm	Before	74.1 ± 10.0	73.4 ± 9.9	74.2 ± 10.2	74.1 ± 9.9
	After	80.8 ± 4.8	79.9 ± 5.6	80.0 ± 4.7	81.6 ± 5.1

Results compare the acoustic baseline against the best-performing fusion model (Acoustic + wav2vec 2.0 + WavLM) across two physiological conditions: resting state (before) and post-exercise (after). Metrics reported as mean ± STD across 5-fold cross-validation.

The combination of acoustic features with wav2vec 2.0 and WavLM emerged again as the best-performing model for this task. In the resting state, the fusion model achieved an F1-score of 74.1 ± 10.0%, together with an accuracy of 73.4 ± 9.9%, a recall of 74.2 ± 10.2%, and a precision of 74.1 ± 9.9%. After exercise, performance increased to an F1-score of 80.8 ± 4.8%, with an accuracy of 79.9 ± 5.6%, a recall of 80.0 ± 4.7%, and a precision of 81.6 ± 5.1%. This corresponds to absolute improvements of 6.7% in F1-score, 6.5% in accuracy, 5.8% in recall, and 7.5% in precision under post-exercise conditions. This behavior can be observed in Figure 2, highlighting the consistent advantage of the SSL-enhanced models over the acoustic baseline. Similarly to the phonation task, the variability of the SSL-enhanced cough model was substantially reduced in the post-exercise condition. For the F1-score, the STD decreased from 10.0% to 4.8%. A comparable stabilization effect was observed for accuracy (9.9% to 5.6%), recall (10.2% to 4.7%), and precision (9.9% to 5.1%).

This stabilization trend is further highlighted when comparing the multi-modal architecture against standalone speech representations. As shown in Table 4, which outlines the performance of individual SSL models in isolation, wav2vec 2.0 alone achieved an F1-score of 77.8 ± 4.5% at rest and 74.7 ± 7.1% post-exercise, whereas WavLM alone reached 75.8 ± 11.4% and 72.7 ± 6.5%, respectively. Much like the phonation task, these isolated models suffer from high performance variability across partitions, exemplified by WavLM's 11.4% STD before exercise. By unifying the acoustic baseline with both SSL extractors, the proposed fusion system simultaneously achieves superior mean F1-scores and tighter confidence intervals in post-exercise conditions, establishing greater diagnostic robustness for cough-based screening.

**Table 4:** Classification performance for the voluntary cough task using individual SSL feature sets (WavLM and wav2vec 2.0) across resting state (before) and post-exercise (after) conditions.

Feature set	Audio moment	F1-score (%)	Accuracy (%)	Recall (%)	Precision (%)
wavlm	Before	75.8 ± 11.4	75.4 ± 11.7	75.6 ± 11.7	76.0 ± 11.1
	After	72.7 ± 6.5	72.8 ± 7.2	72.5 ± 6.3	72.9 ± 6.8
wav2vec	Before	77.8 ± 4.5	77.2 ± 4.1	77.3 ± 4.2	78.3 ± 5.0
	After	74.7 ± 7.1	74.7 ± 7.0	74.5 ± 7.2	75.0 ± 7.1

Metrics are reported as mean ± STD across 5-fold cross-validation and expressed as percentages.

### 3.3 Cross-task model aggregation results

To further explore the complementarity between tasks and recording conditions, a late fusion model aggregation strategy was implemented by combining predictions across vowel phonation (/a/) and cough tasks. As detailed in Table 5, we report the results corresponding to the best-performing configuration (acoustic features combined with wav2vec 2.0 and WavLM embeddings). Aggregation was performed at the subject level using a majority-voting scheme over model outputs, thereby integrating information across tasks and physiological conditions into a unified decision.

**Table 5:** Performance metrics for the cross-task model aggregation strategy using the Acoustic + wav2vec 2.0 + WavLM configuration.

Fusion	Models included		F1-score (%)	Accuracy (%)	Recall (%)	Precision (%)
	Tasks	Audio moment(s)				
Models before	/a/ & cough	Before	81.8	81.8	75.0	90.0
Models after	/a/ & cough	After	84.8	83.8	83.3	86.4
All models	/a/ & cough	Before & after	87.7	87.0	84.5	91.0

Results represent subject-level classification achieved through a majority-voting scheme.

**Figure 3:**
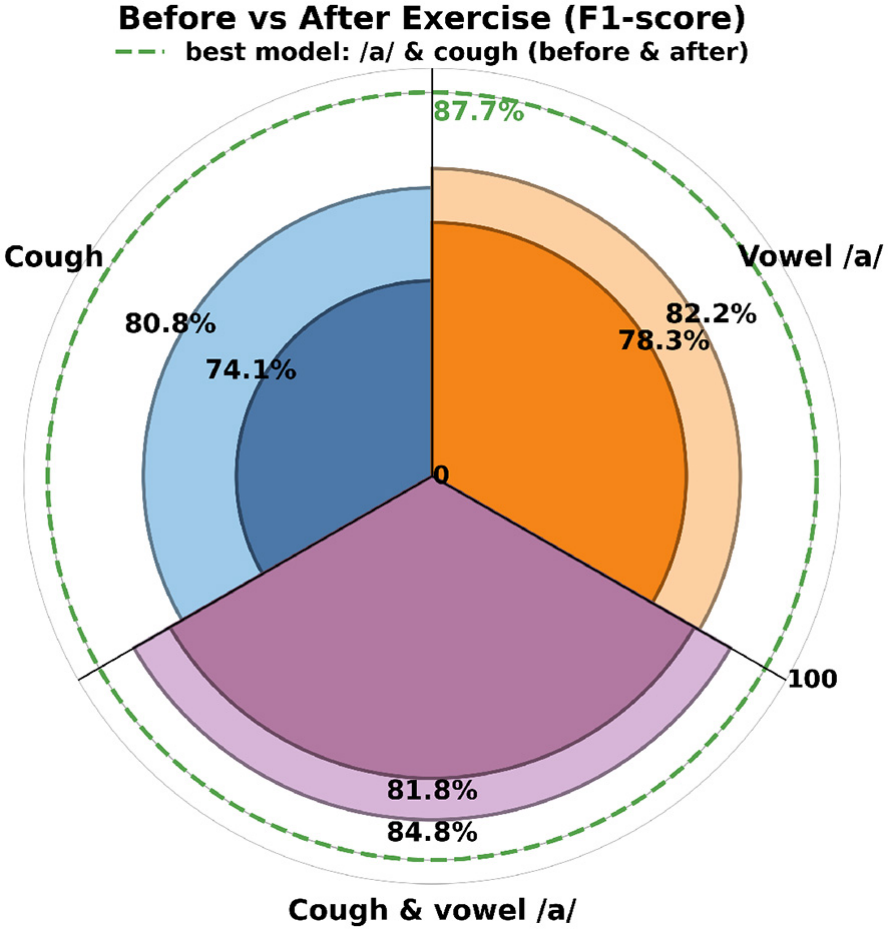
Impact of physical exertion and task aggregation on classification performance (F1-score). The lobes illustrate the performance shift from resting state (before) to post-exercise (after) for the SSL-enhanced models. The dashed green circumference represents the peak system performance (F1-score = 87.7%) achieved through the global late-fusion aggregation of all tasks and physiological conditions.

When aggregating the models trained exclusively on pre-exercise recordings for both tasks, the system achieved the highest pre-exercise F1-score of 81.8%, with an accuracy of 81.8%, a recall of 75.0%, and a precision of 90.0%. Aggregating the post-exercise models yielded an F1-score of 84.9%, accompanied by an accuracy of 83.8%, a recall of 83.3%, and a precision of 86.4%. This result surpasses the strongest individual post-exercise configuration, achieved for sustained vowel /a/ phonation after exercise (F1-score = 82.2%), and also improves the cough task after exercise (F1-score = 80.8%). Finally, the global aggregation which includes all models (both tasks and both recording conditions) achieved the highest overall performance, with an F1-score of 87.7%, an accuracy of 87.0%, a recall of 84.5%, and a precision of 91.0%. This value exceeds all single-task and single-condition models evaluated in this study, including the best standalone result of 82.2% F1-score for post-exercise vowel phonation and 80.8% F1-score for post-exercise cough. Figure 3 visually summarizes this progression, showing the improvement from resting to post-exercise conditions and the highest performance reached through global late-fusion aggregation.

To further assess whether the observed classification performance reflects PASC-specific alterations rather than general physiological or demographic effects, Table 6 reports stratified results across gender and age subgroups for each cross-task aggregation configuration. Performance remained broadly consistent across all subgroups and conditions. For the pre-exercise aggregation, female participants achieved an F1-score of 82.9% and male participants 79.1%, a modest difference that diminished in the combined pre- and post-exercise configuration, where male participants (88.4%) reached a marginally higher F1-score than female participants (87.4%). A similar pattern was observed across age groups: younger participants showed moderately higher performance in the pre- and post-exercise configurations, while both subgroups achieved comparable results in the global aggregation (90.5% vs. 84.6%). Crucially, the post-exercise improvement was consistent across all demographic subgroups, suggesting that the gains attributable to physical exertion are not driven by a uniform confounding response to fatigue or vocal effort, but instead reflect a standardized amplification of PASC-related respiratory and vocal impairments.

**Table 6:** Classification performance stratified by demographic subgroup for the cross-task model aggregation configurations: pre-exercise recordings (before), post-exercise recordings (after), and the global aggregation including both physiological conditions (before & after).

Model fusion		Demographic group		F1-score (%)	Accuracy (%)	Recall (%)	Precision (%)
Tasks	Audio moment(s)	Category	Subcategory				
/a/ & cough	Before	All	-	81.8	81.8	75.0	90.0
		Gender	Female	82.9	81.6	75.4	92.0
		Gender	Male	79.1	82.4	73.9	85.0
		Age group	Younger	85.0	84.0	81.0	89.5
		Age group	Older	78.4	79.7	69.0	90.6
/a/ & cough	After	All	-	84.8	83.8	83.3	86.4
		Gender	Female	86.0	83.5	85.2	86.7
		Gender	Male	81.8	84.3	78.3	85.7
		Age group	Younger	86.7	85.3	85.7	87.8
		Age group	Older	82.9	82.3	81.0	85.0
/a/ & cough	Before & after	All	-	87.7	87.0	84.5	91.0
		Gender	Female	87.4	85.4	85.2	89.7
		Gender	Male	88.4	90.2	82.6	95.0
		Age group	Younger	90.5	89.3	90.5	90.5
		Age group	Older	84.6	84.8	78.6	91.7

Performance is shown for the full cohort and across gender and age group categories.

### 3.4 Statistical validation and model robustness

To assess whether the observed classification performance reflects a genuine, physiologically grounded discriminative signal rather than overfitting to the training data, a *post-hoc* permutation test was conducted for each experimental configuration using 1000 random label permutations. The original and permuted F1-scores, along with their empirical p-values, are detailed in Table 7. Under label permutation, where the relationship between the speech features and the target classes is broken, all models converge to mean F1-scores near the random chance level of 50.2% to 55.3% confirming that the actual observed performance is driven strictly by the intrinsic physiological structure of the data rather than by the model architecture or the regularization scheme alone. For our peak-performing configuration, the acoustic + wav2vec 2.0 + WavLM fusion model post-exercise, we obtained highly significant empirical *p*-values of p = 0.00280 for the vowel phonation task and *p* = 0.00300 for the voluntary cough task, indicating that these results are statistically robust. In comparison, the resting-state acoustic-only models do not reach conventional significance thresholds, yielding non-significant *p*-values of *p* = 0.08172 for vowel phonation and *p* = 0.13307 for voluntary coughing. This systematic drop in p-values from resting acoustic baselines to post-exercise fusion models may align with our hypothesis that baseline resting measurements alone are insufficient to screen for the subtle cardiorespiratory sequelae of PASC, and that the physical exertion protocol is required to act as a physiological provocateur that unmasks these latent markers.

**Table 7:** Permutation test results across tasks, recording conditions, and feature configurations using 1,000 permutations.

Model configuration			Original F1-score (mean, %)	Permuted F1-score (mean ± STD, %)	Permutation test *p*-value
Task	Audio moment	Feature set			
/a/	Before	Acoustic	66.7	51.1 ± 7.2	0.08172
Cough	Before	Acoustic	70.4	51.8 ± 4.3	0.13307
/a/	After	Acoustic	73.6	55.3 ± 4.8	0.05115
Cough	After	Acoustic	77.8	53.3 ± 6.3	0.01099
/a/	Before	Acoustic + wav2vec + wavlm	79.6	52.5 ± 6.7	0.03956
Cough	Before	Acoustic + wav2vec + wavlm	73.7	50.2 ± 4.5	0.05554
/a/	After	Acoustic + wav2vec + wavlm	82.5	53.0 ± 5.8	0.00280
Cough	After	Acoustic + wav2vec + wavlm	80.0	52.1 ± 8.8	0.00300

Original and permuted F1-scores are reported together with the standard deviation of the permuted scores and the corresponding *p*-value.

**Figure 4:**
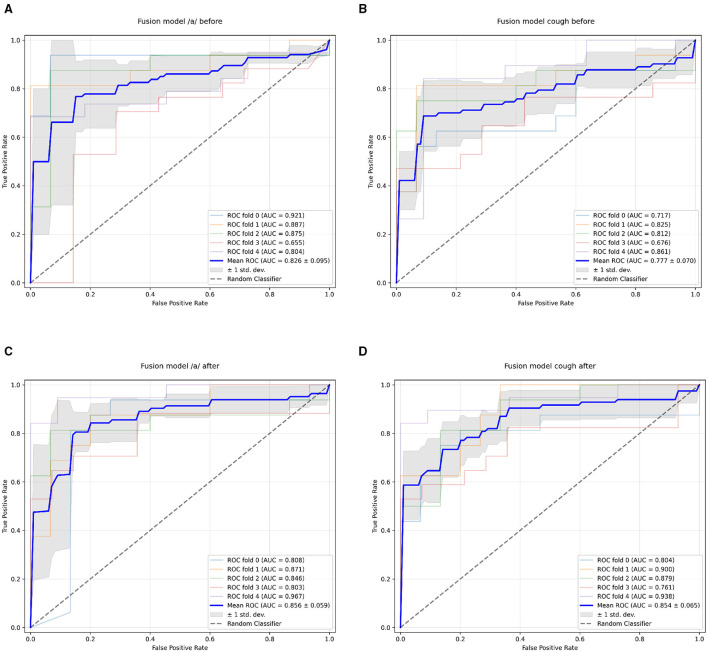
ROC curves for the fusion model (Acoustic + wav2vec 2.0 + WavLM). **(A)** Voluntary cough at rest. **(B)** Sustained vowel /a/ at rest. **(C)** Voluntary cough after exercise. **(D)** Sustained vowel /a/ after exercise. Individual fold ROC curves are shown together with the mean ROC curve and the corresponding ±1 standard deviation interval. The dashed diagonal indicates random classifier performance.

To provide a threshold-independent evaluation of this screening framework, the Area Under the Curve (AUC) values and their 95% Confidence Intervals (CI) across all configurations are reported in Table 8, and the cross-validated Receiver Operating Characteristic (ROC) curves are visualized in Figure 4. As shown in the table, our post-exercise fusion models achieve a substantial expansion in diagnostic capacity, reaching a mean AUC of 85.6 ± 5.94 (95% CI [76.3%; 88.1%]) for vowel phonation and 85.4 ± 6.48 (95% CI [61.7%; 86.5%]) for coughing, compared to resting-state AUC values of 82.6 ± 9.46 (95% CI [62.5%; 94.1%]) for vowel phonation and 77.8 ± 7.01 (95% CI [56.0%; 82.9%]) for cough, respectively. The 95% CIs reported in brackets throughout our tables were computed using the classical t-distribution with 4 degrees of freedom, the critical t-value was set at *t*_crit_ = 2.776, and the margin of error was calculated using the Equation 5:

$$\begin{array}{l} 95\%\text{CI}=\bar{x}\pm \left(t_{\text{crit}}\cdot \frac{s}{\sqrt{n}}\right) \end{array}$$ (5)

where $\bar{x}$ represents the cross-validated mean score and s is the standard deviation across the five folds.

**Table 8:** Summary of AUC, feature-level statistical significance, and Pearson correlation across tasks, recording conditions, and feature configurations.

Model configuration			AUC (mean ± STD) [95% CI]	Pearson correlation (mean ± STD)
Task	Audio moment	Feature set		
/a/	Before	Acoustic	71.8 ± 7.84 [69.5; 84.6]	73.4 ± 3.65
Cough	Before	Acoustic	77.2 ± 9.93 [60.9; 72.4]	75.5 ± 5.27
/a/	After	Acoustic	77.1 ± 4.49 [74.8; 86.8]	76.5 ± 2.85
Cough	After	Acoustic	78 ± 5.71 [63.7; 77.6]	73.7 ± 3.11
/a/	Before	Acoustic + wav2vec + wavlm	82.6 ± 9.46 [62.5; 94.1]	77.8 ± 2.23
Cough	Before	Acoustic + wav2vec + wavlm	77.8 ± 7.01 [56; 82.9]	76.3 ± 3.83
/a/	After	Acoustic + wav2vec + wavlm	85.6 ± 5.94 [76.3; 88.1]	80 ± 2.32
Cough	After	Acoustic + wav2vec + wavlm	85.4 ± 6.48 [61.7; 86.5]	77.2 ± 2.11

Metrics are reported for acoustic and SSL-enhanced models before and after exercise.

Mathematically, narrower confidence intervals indicate high parameter stability and consistent diagnostic effect sizes across patient partitions. In this study, the post-exercise fatigue fusion models achieved the narrowest CIs, suggesting that the physical stress protocol standardizes the physiological states of both PASC patients and healthy controls,thereby improving the consistency of the feature representations and reducing sensitivity to cohort-specific variability.

To visually demonstrate the stability of our LR classifier in this high-dimensional, low-sample-size setting, the figure plots the ROC curves of all five individual cross-validation folds as thin, semi-transparent lines alongside the bold, thick Mean ROC curve and a shaded variance band representing ± 1 standard deviation. Resting conditions are shown in Figures 4A, B; post-exercise conditions in Figures 4C, D. ROC curves demonstrate that post-exercise fusion models maintain high true positive rates across a broad range of False Positive Rates (FPR), with the curve rising steeply in the high-specificity region (FPR below 0.3) that is most relevant for screening applications. While minor dips below the random baseline are observed at very high FPR (greater than 0.9), this is a known artifact in moderate-AUC models with finite sample sizes and reflects limited separation for a small subset of borderline cases under extremely lenient thresholds. Moreover, the ROC curves of the post-exercise fusion models rise toward the upper-left corner of the ROC space, indicating that high sensitivity can be achieved while maintaining relatively low false-positive rates. This behavior is particularly desirable in screening-oriented clinical systems, where minimizing missed pathological cases is critical while still avoiding excessive false alarms. In contrast, the resting-state acoustic-only models exhibit flatter ROC trajectories and wider fold-to-fold variability, suggesting weaker and less stable class separation. The reduced dispersion of the post-exercise ROC curves across folds further indicates that the discriminative boundary learned by the classifier is reproducible across independent subject partitions rather than being driven by specific subsets of patients.

Alongside global performance metrics, we examined the reproducibility of the model's internal feature weights across the five cross-validation folds. For each configuration, the 25 features with the highest mean absolute coefficient were analysed. All of these top-25 features exhibited sign consistency (the same positive or negative sign in at least 4 of the 5 folds), with mean sign agreement of 100% (perfect consistency) in all configurations except one (98.4%). Their median coefficient of variation ($CV=\frac{\text{STD}}{\left|\text{mean}\right|}$) was low, ranging from 0.12 to 0.28 across configurations, and 92-100% of these top predictors had a CV below 0.5. This demonstrates that the features the model actually relies on are assigned highly reproducible weights, ruling out the possibility that the classifier merely memorizes partition-specific noise.

To further substantiate the interpretability of the linear model, as shown in Table 9, we intersected the top-25 most important features selected using the absolute mean value of the coefficient with those that pass the strict univariate False Discovery Rate (FDR) multiple-testing correction (*p*_*FDR*_ < 0.05), have a *CV* < 0.5 and maintain the same sign in all the folds. In the best post-exercise vowel fusion model, 15 features satisfied all four criteria: high coefficient magnitude, sign consistency, low CV, and FDR-significant univariate separation. These 15 features span three temporal audio slots (task repetitions), capturing the dynamic progression of vocal production. Specifically, they comprise aggregated statistics from classical acoustic biomarkers that align with established clinical markers: MFCCs contribute measures of intra-utterance variability and resonance stability (e.g., standard deviation and kurtosis), alongside quartile and extrema distributions that track spectral envelope asymmetry and phonation consistency. RASTA-filtered spectral features leverage quartile and minimum/maximum statistics to suppress channel noise while emphasizing relative spectral shifts indicative of formant stability. Acoustic energy quartiles reflect phonatory power and intensity control, while basic spectral descriptors incorporate entropy and minimum values to quantify spectral disorder and purity. By summarizing both central tendency and variance structure across these aggregated statistics, the model may detect subtle pathological patterns that single-metric approaches might overlook. Furthermore, the temporal distribution across the three audio slots enables the identification of fatigue accumulation, phonatory consistency, and recovery dynamics, which are indicators of vocal function. Finally, WavLM self-supervised embeddings complement these classical biomarkers by encoding latent, data-driven acoustic patterns learned from large-scale speech corpora. Together, this multi-faceted feature set could show that disease-related acoustic alterations–whether manifesting as tremor, hoarseness, reduced stability, or spectral noise–are reliably detected through both physiologically grounded parameters and representation-learned cues, relating model decisions to meaningful vocal physiology. The remaining 10 top features, while not individually significant at the univariate level, were equally stable in sign and magnitude, indicating that the model integrates subtle, distributed acoustic alterations that are invisible to simple group comparisons. Many of these were SSL embedding dimensions, highlighting their complementary role. For our post-exercise cough fusion model, 18 features pass the FDR-corrected Mann-Whitney threshold, while 6 features do not. This four-way intersection analysis shows that a substantial subset of the learned biomarkers remains both statistically and structurally stable across folds.

**Table 9:** Feature stability and consistency analysis across tasks, recording conditions, and feature configurations.

Model configuration			Median CV (Top 25 features)	Stable features in Top 25 (CV < 0.5, %)	Mean sign consistency (%)	Top 25 features meeting all selection criteria	Top 25 features meeting consistency and stability criteria
Task	Audio moment	Feature set					
/a/	Before	Acoustic	0.186	100	100	21	4
Cough	Before	Acoustic	0.284	96	100	8	16
/a/	After	Acoustic	0.167	100	100	22	3
Cough	After	Acoustic	0.256	92	98.4	13	10
/a/	Before	Acoustic + wav2vec + wavlm	0.152	100	100	22	3
Cough	Before	Acoustic + wav2vec + wavlm	0.207	100	100	13	12
/a/	After	Acoustic + wav2vec + wavlm	0.123	100	100	15	10
Cough	After	Acoustic + wav2vec + wavlm	0.159	96	100	18	6

Metrics summarize variability, sign consistency, and the number of top-ranked features meeting the defined selection criteria.

Finally, Table 8 also reports the Pearson correlation between model predictions and true labels, providing a continuous-output measure of association complementary to the threshold-dependent F1-score. Pearson correlation coefficients range from 0.734 ± 0.037 for the resting acoustic vowel model to 0.80 ± 0.023 for the post-exercise fusion vowel model, with the latter representing a large effect size according to conventional benchmarks. Unlike the F1-score, which depends on a fixed decision threshold, the Pearson correlation captures the strength of the linear relationship between the classifier's continuous confidence scores and the binary diagnostic outcome, thereby offering a threshold-free view of discriminative capacity. The post-exercise fusion models not only achieved the highest mean Pearson correlations (vowel: 0.80 ± 0.023; cough: 0.772 ± 0.021) but also exhibited the smallest standard deviations across folds, consistent with the variance reduction observed for the F1-score and AUC. This convergence of threshold-dependent and threshold-independent metrics reinforces the conclusion that the exercise-enhanced model does not merely memorize a decision boundary that works for one particular train-test split; rather, it learns a robust and reproducible mapping from voice features to PASC status. The monotonic increase in Pearson correlation from acoustic-only to fusion and from resting to post-exercise conditions further supports the complementary nature of the SSL embeddings and the diagnostic value of the physical stress protocol.

## 4 Discussion

The experimental findings of this study demonstrate that automatic detection of PASC can be significantly improved through two complementary strategies: (I) the incorporation of controlled physical exertion at the recording stage and (II) the integration of traditional acoustic features with high-dimensional SSL embeddings. Across all experiments, metrics such as accuracy, recall, and precision followed trends similar to the F1-score; consequently, we focus this section on the F1-score, as it provides a balanced representative of overall model performance.

### 4.1 Physical exertion as diagnostic enhancer

The core contribution of this research lies in the validation of fatigue-induced signal analysis for the detection of PASC. For both sustained phonation and coughing tasks, the post-exercise condition consistently yielded higher classification metrics compared to baseline measurements. Specifically, the optimal model achieved its peak performance after exertion, reaching an F1-score of 82.2% for phonation and 80.8% for coughing. Although prior studies have noted that physical stress exacerbates acoustic irregularities (29, 30), our findings extend this observation to voice-based detection of PASC.

A comparative evaluation across tasks and physiological conditions reveals several key insights into the behavior of voice signals as biomarkers of PASC. When examining the sensitivity of each task to fatigue, the cough task demonstrated a more pronounced sensitivity than the sustained vowel task. Within the acoustic baseline, the cough task showed an absolute improvement in the F1-score of 7.6%, nearly double the absolute improvement of 4.0% seen in the sustained vowel task. This sensitivity to exercise was also evident when the fusion of wav2vec 2.0, WavLM, and acoustic features was employed. This suggests that the respiratory mechanics and rapid expiratory flow required for coughing are more immediately impacted by the physical exertion of the six-minute walk test than the phonatory control required for vowel production.

The higher sensitivity of the cough signal to physical stress can be explained by its physiological nature. Unlike the sustained vowel, which relies on glottal vibration, a cough is a complex, multi-phase event defined as a deep inspiration followed by complete glottal closure, a forced expiratory effort generating high pressure, and subsequent glottal opening with rapid expiratory airflow (31). Because PASC is associated with respiratory muscle fatigue and dyspnea, physical exertion likely functions as a physiological stressor that reveals subclinical impairments in cough effectiveness. This phenomenon is consistent with findings in patients with other respiratory diseases, where impairments in expiratory airflow and delayed achievement of peak expiratory velocities serve as indicators of cough dysfunction (32). Under such fatigue conditions, the reduced ability to generate rapid and forceful expiratory flow can produce measurable acoustic alterations, providing a potential marker for the assessment of cough performance.

Furthermore, physical exertion improved not only the magnitude of the results, but also the robustness of the classification; the STD for the optimal feature set was reduced by more than a half in the post-exercise subsets, as seen in Tables 1, 3. This suggests that the physiological impact of the six-minute walk test and the sit-to-stand task standardizes the manifestations of PASC-related impairment, leading to more stable and reliable ML performance.

While the observation that physiological stress may amplify pathological vocal markers is consistent with established cardiopulmonary principles (33), its clinical relevance lies in supporting a more objective screening approach. PASC is characterized by heterogeneous and frequently subclinical impairments that may not be fully captured during routine resting examinations. In this context, a standardized physiological challenge may help reveal latent respiratory-vocal alterations associated with disease status. In addition, the reduced classification variability observed post-exercise suggests improved biomarker consistency, potentially supporting longitudinal follow-up. Overall, this methodology may offer a reproducible and clinically relevant complement to conventional functional assessment.

### 4.2 Effect of integrating SSL embeddings

The experimental results highlight the strong complementarity between traditional acoustic features and high-dimensional SSL representations. Traditional acoustic features were incorporated as they represent the established standard in pathological voice analysis, providing physiologically interpretable markers that are widely validated in clinical acoustic research and ensure comparability with prior work in the field. Moreover, while acoustic features (e.g., MFCCs, jitter, shimmer) provide a concrete physiological baseline, they are inherently limited by their reliance on predefined mathematical descriptors. In contrast, SSL models like wav2vec 2.0 and WavLM generate high-dimensional latent representations that may provide complementary discriminative information beyond conventional human-engineered features. Using these capabilities, previous work has shown that SSL-based embeddings can enhance the detection of subtle speech impairments in neurological and pathological conditions (34, 35). Extending this perspective, our results indicate that these representations are highly sensitive to respiratory and vocal alterations induced by fatigue in post-viral conditions such as PASC, potentially supporting non-invasive detection and monitoring of these impairments.

The superior performance of the fusion models (acoustic + wav2vec 2.0 + WavLM) suggests a synergistic effect, as this combination achieved the highest results in all experimental scenarios. Specifically, for the sustained vowel task, the fusion model reached an F1-score of 82.2% after exercise, surpassing the acoustic-only baseline of 70.7%. A similar trend was observed in the cough task, where the fusion model peaked at 80.8% compared to the baseline of 77.1%.

The sustained vowel task exhibited a larger relative improvement with the incorporation of SSL embeddings compared to the cough task, as shown in Tables 1, 3, a pattern that is also reflected in Figure 2, which summarizes these differences across tasks and recording conditions. This difference may be attributed to the structural properties of the signals. Sustained phonation is characterized by periodic glottal vibration, providing a stable temporal framework in which subtle perturbations can be effectively modeled by deep contextual architectures. In contrast, cough signals are brief and highly variable events, which may reduce the additional benefit obtained from high-dimensional representation learning beyond what is already captured by conventional acoustic features.

To examine the individual contribution of SSL embeddings, Tables 2, 4 present results for WavLM and wav2vec 2.0 used in isolation. These results confirm that SSL embeddings have discriminative performance, with wav2vec 2.0 alone reaching 80.4 ± 6.6% F1-score for resting vowel phonation. However, individual SSL models exhibit notably high standard deviations in several conditions, most markedly WavLM post-exercise on the vowel task (74.4 ± 15.0%), indicating substantial fold-to-fold variability. In contrast, the fusion of acoustic features with wav2vec 2.0 and WavLM consistently achieves higher mean performance with lower standard deviation in post-exercise conditions (82.2 ± 4.7% and 80.8 ± 4.8% for vowel and cough respectively), indicating that the combination produces a more stable and reliable classifier.

### 4.3 Cross-task synergy

The diagnostic efficacy of voice-based screening depends on the diversity of physiological information captured by the analyzed signals. While sustained vowels represent phonatory stability and vocal fold control, cough signals reflect respiratory airflow dynamics. Integrating these tasks allows for a more comprehensive assessment of the respiratory-vocal system than any single task could provide. This complementarity is further supported by our experimental results, where the integration of multiple tasks consistently outperformed single-task models, as shown in Table 5.

A late fusion aggregation strategy was applied to combine predictions across tasks and physiological states. When aggregating models trained exclusively on pre-exercise recordings, the system achieved an F1-score of 81.8%. This represents an absolute improvement of 3.5% over the fusions models at rest (sustained vowel /a/, F1-score = 78.3%) and 7.7% over the resting cough task (F1-score = 74.1%). Similarly, the aggregation of post-exercise models reached an F1-score of 84.8%, surpassing the strongest post-exercise individual task (sustained vowel /a/, F1-score = 82.2%) by 2.6% and the post-exercise cough task (F1-score = 80.8%) by 4.0%. These results highlight the effectiveness of late fusion in reducing the variability associated with individual tasks. By integrating complementary information from both phonatory and cough tasks, the multi-task approach stabilizes the predictive output and achieves higher performance than any single recording protocol alone, regardless of the patient's physiological condition.

When all tasks and physiological conditions were jointly considered, the aggregated model achieved the best overall performance of the study, reaching an F1-score of 87.7% and surpassing all individual task configurations. This indicates that the highest performance is achieved when the full patient' speech profile is considered, effectively capturing the diverse acoustic manifestations of PASC across tasks that vary in physical and vocal demand, a progression that is also reflected in Figure 3.

The performance improvement observed in the aggregated models provides further insight into the underlying vocal alterations, indicating that PASC-related impairments manifest across multiple dimensions of the respiratory-phonatory system and may not be fully captured by any single vocal task. By combining predictions derived from both tasks, the system effectively integrates information about respiratory airflow dynamics and vocal fold vibration stability. As a result, this aggregated model benefits from a broader physiological perspective of the patient's respiratory and vocal function.

A primary consideration in deploying ML approaches for clinical screening is ensuring model stability and ruling out potential demographic biases or overfitting induced by confounding variables such as age and sex. In this study, this concern is mitigated by our matched cohort design, which controlled for age and sex, and the execution of a standardized physical stress protocol uniform to all participants. Most notably, our stratified results Table 6 show that post-exercise performance gains remain highly consistent across distinct age and sex subgroups. This pattern strongly indicates that controlled physical stress acts as a clinical provocateur that selectively amplifies respiratory and vocal alterations associated with PASC, rather than introducing a uniform confounding effect.

### 4.4 Conclusions

This study demonstrates that the objective detection of PASC is significantly enhanced by integrating physiological stress tests with advanced speech representations. Physical exertion, induced through six-minute walk and sit-to-stand tests, acts as a diagnostic amplifier, yielding post-exercise recordings to improve classification performance and reduce STD. This suggests that physiological stress standardizes pathological manifestations, making latent respiratory and vocal impairments more detectable to ML models. The integration of traditional acoustic features with high-dimensional SSL embeddings, such as wav2vec 2.0 and WavLM, further enhances diagnostic sensitivity by incorporating complementary data-driven speech representations.

Beyond PASC as a case study, the methodological framework proposed here may have a broader applicability. Since the approach focuses on detecting respiratory and vocal impairments through stress-modulated acoustic analysis, it could be extended to other conditions in which physiological stress reveals impairments in respiratory or vocal function, including chronic respiratory diseases and neurological disorders affecting speech production. However, despite the encouraging results of this study, these findings should be interpreted with caution. The sample size, although acquired in a controlled clinical setting, remains moderate and geographically localized. In addition, the use of a linear classifier was intentionally chosen for interpretability; future studies could explore other architectures to further exploit the representational richness of SSL embeddings.

Overall, these results demonstrate the value of integrating physiological stress with acoustic analysis as a non-invasive and scalable strategy for detecting respiratory and vocal impairments. While the amplification of pathological signals under exertion is physiologically expected, incorporating this response into a standardized, low-burden protocol may help bridge the gap between research biomarkers and routine clinical screening. The physical stress protocols employed in this study (the six-minute walk test and one-minute sit-to-stand test) are already widely used in cardiopulmonary rehabilitation and primary care settings, which may facilitate clinical implementation. Positioning PASC as an initial clinical application within a broader framework for respiratory health assessment, this methodology offers a reproducible pathway toward objective, fatigue-modulated voice assessment and may support future screening, follow-up, and personalized rehabilitation across broader respiratory and neurological conditions.

## Data Availability

The datasets presented in this article are not readily available because the dataset used in this study is not publicly available. Requests to access the datasets should be directed to Laura Docío-Fernández, ldocio@gts.uvigo.es.
