# Supplementary material for: Improving respiratory disease detection through SSL-enhanced acoustic analysis and exercise-rest measurements
Source: Front Med (Lausanne). 2026 Jun 24;13:1864436. doi: 10.3389/fmed.2026.1864436 (PMC13342220; doi:10.3389/fmed.2026.1864436)
Supplement: Supplementary file 1 [file Data_Sheet_1.PDF]

Supplementary Material

1 FEATURE EXTRACTION DETAILS

Table S1 summarizes the dimensionality of the feature representations evaluated in this study, including both handcrafted acoustic descriptors and SSL embeddings. Reported values correspond to the fixed-dimensional vector extracted from each individual recording before participant-level concatenation.

Feature	#
energy-based descriptors	132
voicing features	198
RASTA-filtered spectral features	858
basic spectral descriptors	495
MFCCs	429
Wav2vec 2.0 embeddings	1024
WavLM embeddings	768
HuBERT embeddings	768

Table S1. Dimensionality of the feature representations evaluated in this study, including handcrafted acoustic descriptors and SSL embeddings. Reported values correspond to the fixed-dimensional representation extracted from each individual recording.

The statistical aggregation procedure applied to each frame-level descriptor is summarized in Table S2.

Statistic	Description
Mean	Arithmetic average of the temporal trajectory of the feature across frames.
Standard deviation	Measures dispersion or variability of the feature values over time.
Median	Robust central tendency statistic less sensitive to outliers than the mean.
Minimum	Lowest observed feature value within the utterance.
Maximum	Highest observed feature value within the utterance.
Range	Difference between maximum and minimum values, reflecting dynamic span.
Skewness	Measures asymmetry of the feature distribution over time.
Kurtosis	Measures peakedness and tail heaviness of the feature distribution.
First quartile (Q1)	25th percentile of the feature distribution.
Third quartile (Q3)	75th percentile of the feature distribution.
Interquartile range (IQR)	Difference between Q3 and Q1, representing robust variability.

Table S2. Statistical functionals used to aggregate frame-level acoustic descriptors into utterance-level representations.

Table S3 summarizes the dimensionality of the two feature configurations reported in the main manuscript: the handcrafted acoustic baseline and the best-performing fusion model combining acoustic descriptors with Wav2vec 2.0 and WavLM embeddings.

Feature configuration	#
acoustic	2112
acoustic + Wav2vec 2.0 + WavLM	3904

Table S3. Dimensionality of the two feature configurations reported in the main manuscript. Values correspond to the representation extracted for each recording prior to classification.
